# Development of a non-invasive method for skin cholesterol detection: pre-clinical assessment in atherosclerosis screening

**DOI:** 10.1186/s12938-021-00889-1

**Published:** 2021-06-01

**Authors:** Jingshu Ni, Haiou Hong, Yang Zhang, Shiqi Tang, Yongsheng Han, Zhaohui Fang, Yuanzhi Zhang, Nan Zhou, Quanfu Wang, Yong Liu, Zhongsheng Li, YiKun Wang, Meili Dong

**Affiliations:** 1 Anhui Provincial Engineering Technology Research Center for Biomedical Optical Instrument, Anhui Institute of Optics and Fine Mechanics, Hefei Institutes of Physical Science , Chinese Academy of Sciences, Hefei, 230031 China; 2 University of Science and Technology of China, Hefei, 230026 China; 3 Wanjiang Center for Development of Emerging Industrial Technology, Tongling, 244000 China; 4 Health Management Center, First Affiliated Hospital of University of Science and Technology of China, Hefei, China; 5 Health Management Center, Renmin Hospital of WuHan University, Wuhan, 430060 China; 6 Department of Cardiovascular Medicine, First Affiliated Hospital of University of Science and Technology of China, Hefei, 230001 China; 7 Department of Endocrinology, The First Affiliated Hospital of Anhui University of Traditional Chinese Medicine, Hefei, 230031 China

**Keywords:** Non-invasive, Skin cholesterol, Absorption spectroscopy, Subclinical atherosclerosis

## Abstract

**Background:**

Establishing a high-accuracy and non-invasive method is essential for evaluating cardiovascular disease. Skin cholesterol is a novel marker for assessing the risk of atherosclerosis and can be used as an independent risk factor of early assessment of atherosclerotic risk.

**Methods:**

We propose a non-invasive skin cholesterol detection method based on absorption spectroscopy. Detection reagents specifically bind to skin cholesterol and react with indicator to produce colored products, the skin cholesterol content can be obtained through absorption spectrum information on colored products detected by non-invasive technology. Gas chromatography is used to measure cholesterol extracted from the skin to verify the accuracy and reliability of the non-invasive test method. A total of 342 subjects were divided into normal group (*n* = 115), disease group (*n* = 110) and risk group (*n* = 117). All subjects underwent non-invasive skin cholesterol test. The diagnostic accuracy of the measured value was analyzed by receiver-operating characteristic (ROC) curve.

**Results:**

The proposed method is able to identify porcine skin containing gradient concentration of cholesterol. The values measured by non-invasive detection method were significantly correlated with gas chromatography measured results (*r* = 0.9074, *n* = 73, *p* < 0.001). Bland–Altman bias was − 72.78 ± 20.03 with 95% limits of agreement − 112.05 to − 33.51, falling within the prespecified clinically non-significant range. We further evaluated the method of patients with atherosclerosis and risk population as well as normal group, patients and risk atherosclerosis group exhibited higher skin cholesterol content than normal group (all *P* < 0.001). The area under the ROC curve for distinguishing Normal/Disease group was 0.8642 (95% confidence interval, 0.8138 to 0.9146), meanwhile, the area under the ROC curve for distinguishing Normal/Risk group was 0.8534 (95% confidence interval, 0.8034 to 0.9034).

**Conclusions:**

The method demonstrated its capability of detecting different concentration of skin cholesterol. This non-invasive skin cholesterol detection system may potentially be used as a risk assessment tool for atherosclerosis screening, especially for a large population.

**Supplementary Information:**

The online version contains supplementary material available at 10.1186/s12938-021-00889-1.

## Background

Cardiovascular disease is the leading cause of death worldwide, atherosclerosis constitutes the main pattern of cardiovascular disease. Effective control at an early stage will delay or prevent the development of asymptomatic atherosclerosis into cardiovascular disease [1–3]. At present, for the detection of atherosclerosis, examinations such as angiography and ultrasound can detect abnormalities only after the lesions have appeared in the arteries, thus there are limitations in the early screening of atherosclerosis. Other risk factors such as blood lipids, blood sugar and other indicators are invasive tests, and are affected by factors such as diet and fluctuation. There is an urgent need for clinical non-invasive and convenient atherosclerosis early screening methods.

The skin barrier is located in the uppermost layer of the skin, the stratum corneum. Its function is to protect the body from excessive trans-epidermal water loss (TEWL), as well as to prevent the penetration of compounds into the body via the epidermis. The main lipid classes of human stratum corneum are composed of ceramides, free fatty acids and cholesterol, which makes skin an active site of cholesterol synthesis [4–6]. For most of the previous studies, cholesterol synthesis is mainly essential for skin barrier homeostasis [7–9], a recent research has reported the impact of cholesterol depletion on the permeability properties and microstructure of model membranes and human, they speculate that the stratum corneum cholesterol domains may have a more complex role in the skin, other than a barrier limiting water loss and the entry of chemicals [10]. Numerous studies have shown that skin cholesterol (SC) content is associated with deposition of cholesterol in the coronary arteries and aorta [11–15]. Thus, SC content has been suggested to being quantified as a marker for identifying patients with atherosclerotic arterial disease. Previous efforts in larger studies of SC were hampered by methods that required a skin sample for cholesterol measurement, however, these concerns have now been resolved by a non-invasive method to determine SC for cardiovascular risk assessment [16, 17].

Using above assay that measures SC content of epidermis, SC is proved to be associated with angiographic coronary artery disease, and the presence of myocardial ischemia in patients with positive stress tests results. In asymptomatic patients, there is an association between SC and coronary artery calcium and circulating inflammatory markers and with CIMT [18–20]. All these studies reveal that SC may be a marker for subclinical atherosclerosis, and SC content could become a useful tool for assessing cardiovascular risk, which means it is capable of discriminating among healthy individuals, those at risk of developing atherosclerosis, and those with overt disease. The above non-invasive measurement of SC content use a point-of-care test defined as Cholesterol 1,2,3™, which contains three different concentrations of HRP conjugate used to bind to cholesterol in the skin and then visual scoring by the indicator. A hand-held instrument was used to carry out in situ detection of the diffuse reflection spectrum of the indicator. The light passes through the detection reagent, reaches the skin epidermis, and then returns to the detector. The light reflected back to the detector not only contains the information of the indicator, but also the absorption and scattering effects from skin of different people, therefore, the measurement results are affected by the absorption and scattering substances in the skin [21, 22]. Meanwhile, the influence of uncontrolled pressure on the physiologic status of the skin also affects the diffuse reflectance, which may lead to fluctuations in the measurements of different people [23]. Thus, the measurement is easily affected by the pressure change caused by hand-held instrument operated by different operators, besides, the absorption and scattering substances of different palms from different population differs from each other which may lead to measured deviation when in situ measurement is performed.

A reasonable approach to tackle these issues is to perform No-Touch palm measurement utilizing absorption spectrum instead of diffuse reflection spectrum. Strategy of No-Touch palm measurement would make it overcome the pressure change caused by different operator; meanwhile, the method can be more accurate by absorption spectrum regardless of absorption and scattering substances difference of different population’s palm. To this end, we develop a No-Touch palm measurement device, which can non-invasively assess cholesterol in epidermis, and the following study is to introduce our non-invasive skin cholesterol detection system and prove the accuracy and stability of the device as well as exploring the initial clinical application in the Chinese population.

## Results

### Non-invasive skin cholesterol detection system is capable of recognizing gradient color solution

**Fig. 1:**
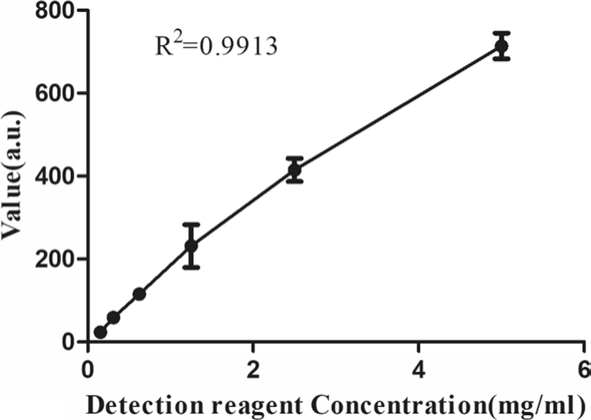
The fitted curve of different gradation of color induced by TMB and detection reagent. TMB catalyzed by 5 μg/ml, 2.5 μg/ml, 1.25 μg/ml, 0.625 μg/ml, 0.3125 μg/ml and 0.15625 μg/ml of the detection reagent, respectively, the amount of colored products produced by the reaction was measured with our system. The values are presented as the means ± the SDs of three independent repeated experiments

The final variations in the color of blue chromogenic reagent catalyzed by detection reagent linked to skin cholesterol should be recognized by our device. The amount of colored products produced by the reaction between the substrate and the enzyme is non-linear, so we calibrate the standard curve of colored product with the instrument. Gradient concentration of detection reagent was reacted to excess mount of TMB to simulate the reaction between cholesterol and detection reagents, as shown in Fig. 1, the larger value of reaction product detected by our device is as the detection reagent concentration increases. The fitted curve obtained by the experiment can be used as a calibration curve to calculate the concentration information of the cholesterol content in the skin sample to be tested.

### Non-invasive skin cholesterol detection system can distinguish gradient skin cholesterol in pig skin

**Fig. 2:**
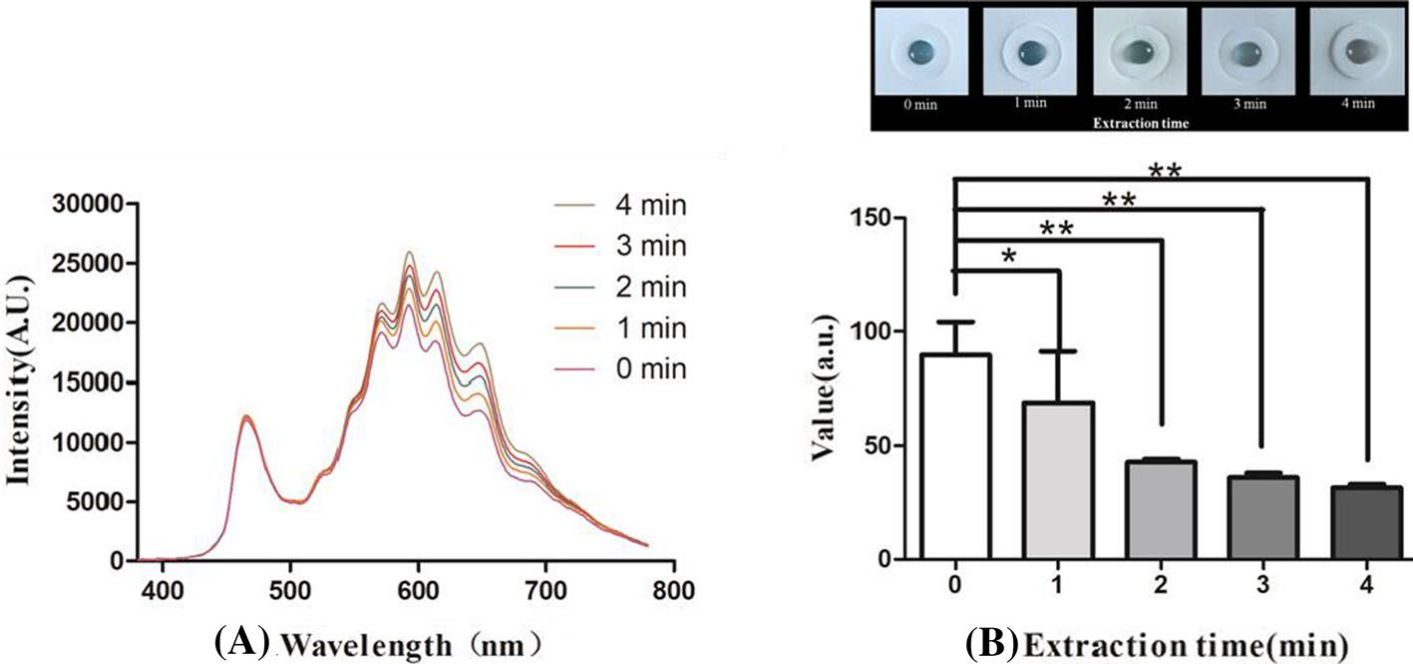
No-Touch palm measurement device can distinguish gradient skin cholesterol in pig skin extracted with the mixture of ethanol and ethyl ether with a proportion of 3:1 for different time course. **A** The absorption spectroscopy under different extraction time; **B** the variation of values with the increased extracting time

To mimic the measurement of skin cholesterol in different humans, pig skin extracted with the mixture of ethanol and ethyl ether with a proportion of 3:1 for different time course (0 min, 1 min, 2 min, 3 min, 4 min) was used to obtain skin containing gradient cholesterol. As shown in Fig. 2A, B, with the increase of extraction time, the shape of absorption spectrum remained unchanged and the intensity gradually increased and the color of blue become lighter, meanwhile, the value measured by device is decreased with the prolongation of extraction time (Fig. 2B).

### Accuracy and reliability analysis of non-invasive skin cholesterol detection

**Fig. 3:**
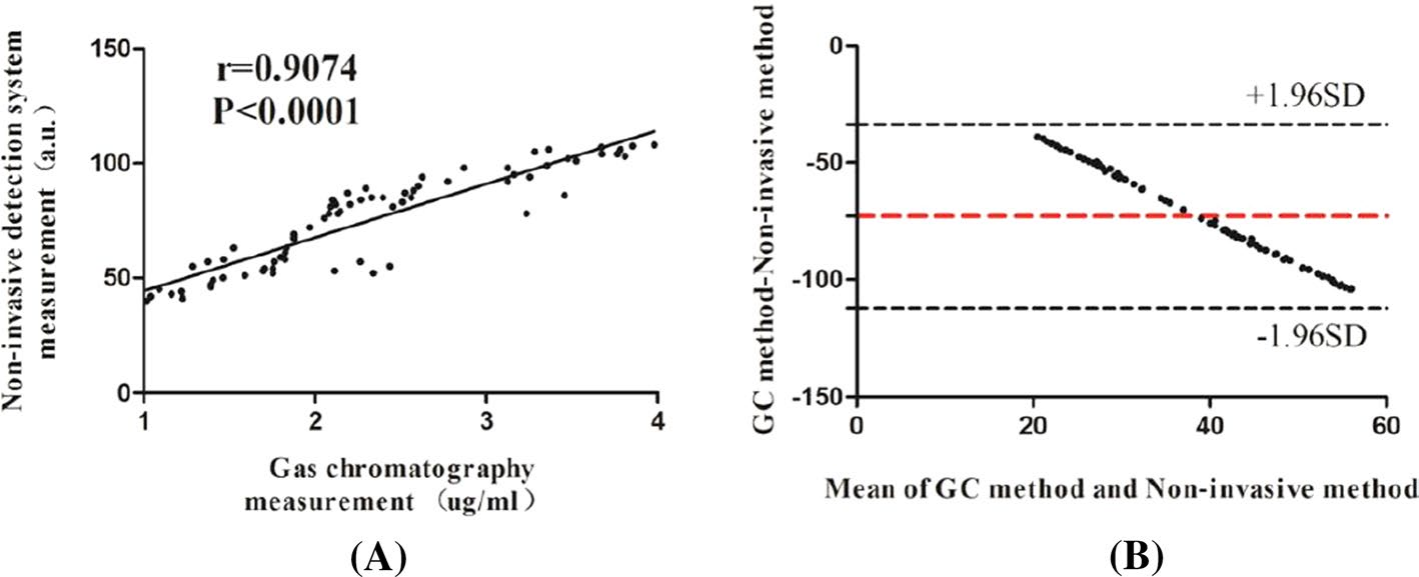
Accuracy and reliability analysis of non-invasive skin cholesterol detection. **A **The correlation between skin cholesterol content measured by gas chromatography and non-invasive detection method. **B **Bland–Altman analysis of the results detected by non-invasive method and the values measured by gas chromatography

To determine the accuracy of non-invasive skin cholesterol detection system, we extract the cholesterol in epidermis after non-invasive measurement with our detection system, and the cholesterol in extractive liquid was measured by gas chromatography. Correlation between skin cholesterol content measured by gas chromatography and non-invasive detection method was analyzed. As shown in Fig. 3A, the correlation coefficient was 0.9074, and there is a prominently strong correlation between the non-invasive detection system measured value and gas chromatography measured value. Meanwhile, we have compared the results detected by non-invasive method with the values measured by gas chromatography using the Bland–Altman analysis. Figure 3B shows that the Bland–Altman bias was − 72.78 ± 20.03 with 95% limits of agreement − 112.05 to − 33.51, falling within the prespecified clinically non-significant range. The results indicated that the non-invasive method is a reliable and accurate method for the detection of skin cholesterol.

### Non-invasive skin cholesterol detection system can distinguish subclinical atherosclerosis, atherosclerosis patients and healthy individuals

**Fig. 4:**
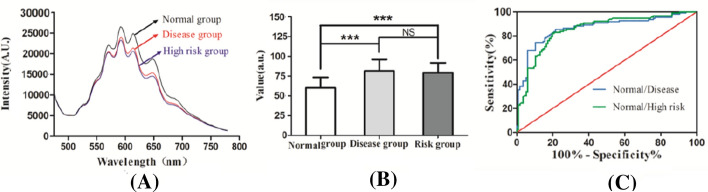
No-Touch palm measurement device can distinguish subclinical atherosclerosis, atherosclerosis patients and healthy individuals. **A** The absorption spectroscopy of normal group, disease group and high-risk group. **B** Skin cholesterol values of normal group, disease group and high-risk group detected by non-invasive measurement system. **C** Receiver-operating characteristic (ROC) curves for distinguishing Normal/Disease group and Normal/High-risk group

To examine whether No-Touch palm measurement device can recognize healthy individuals and atherosclerosis patients, as well as high-risk atherosclerosis population, 110 atherosclerosis patients and 117 high-risk populations were measured, meanwhile, 115 low-risk individuals were also enrolled as normal groups. Detailed subject characteristic is shown in Table 1. The result revealed that the shape of absorption spectrum remained the same, the intensity of the normal group is stronger than that of disease group and high-risk group (Fig. 4A). Meanwhile, disease group and high-risk group have a significant higher skin cholesterol value compared to normal group (Fig. 4B), however, the values between disease group and high-risk group did not have any significant difference. The area under the ROC curve was applied to evaluate the efficacy of skin cholesterol values on screening for atherosclerosis risk. As shown in Fig. 4C, the area under the ROC curve for distinguishing Normal/Disease group was 0.8642 (95% confidence interval, 0.8138 to 0.9146), meanwhile, the area under the ROC curve for distinguishing Normal/Risk group was 0.8534 (95% confidence interval, 0.8034 to 0.9034). The efficacy of skin cholesterol values for distinguishing Normal/High-risk group is similar to that of Normal/Disease group.

**Table 1:** Subject characteristic (*n* = 342)

Variable	Normal group	Risk group	Disease group
N	115	117	110
Female (%)	39 (33.91%)	42 (35.90%)	36 (32.73%)
Age (yrs ± SD)	50.33 ± 10.12	52.28 ± 12.81	53.16 ± 12.13
BMI (kg/m^2^ ± SD)	26.31 ± 3.12	26.59 ± 5.93	26.13 ± 4.29
History of diabetes mellitus	7 (6.09%)	15 (12.82%)	12 (10.91%)
History of hypertension	26 (22.61%)	39 (33.33%)	37 (33.64%)
Current smoker	35 (30.43%)	41 (35.04%)	36 (32.73%)
Framingham score (%)	8.3 ± 3.3	17.12 ± 5.21^**^	19.23 ± 5.32^**^
TC (mmol/L)	4.37 ± 0.75	5.41 ± 0.81^*^	5.38 ± 0.49^*^
LDL-C (mmol/L)	3.25 ± 0.93	3.58 ± 0.98^*^	3.49 ± 0.72^*^
HDL-C (mmol/L)	0.91 ± 0.20	0.91 ± 0.31	0.90 ± 0.16
TG (mmol/L)	1.52 ± 0.36	1.61 ± 0.51	1.63 ± 0.39

Continuous values are presented as mean ± SD, categorical values are presented as number of patients (percentage)

^*^P < 0.05, ^**^P < 0.01 vs. the normal group

## Discussion

In this study, we proposed a No-Touch palm measurement device for non-invasive skin cholesterol detection. Compared to the reported in situ detection used in the previous clinical research, the proposed device may offer more accurate results. The previously reported diffuse reflection method requires the operator to carry out in situ detection with a hand-held instrument. The light passes through the detection reagent, reaches the skin epidermis, and then returns to the detector. Due to the different content of absorbed and scattered substances in the skin from different races [22, 24, 25], the light reflected back to the detector not only contains the information of the detection reagent, but also the absorption and scattering effects from skin of different people. However, our method does not require in vivo measurement, we move the reaction solution to the sample platform, all the detection signal is from the reaction solution, there is no interference from skin background. Meanwhile, uncontrolled probe-to-tissue coupling and pressure can make it difficult to obtain a reproducible spectrum, particularly for an untrained operator. There was a decrease in the diffuse reflectance and an increase in the scattering coefficient between 400 and 1800 nm with compression of in human skin [23]. When in situ detection is performed by a hand-held instrument, the pressure is easily affected by different operators, and at the same time, it is also affected by the tightness of the measurement part of the subject. Our device uses non-touch palm measurement, which can avoid the influence of the above two factors on the measurement results. Therefore, this method may be more suitable for studying the relationship between skin cholesterol and atherosclerosis in different races around the world.

For optical measuring device, the stability of the light source is critical to the accuracy of the measurement. The simulation experiment result showed that light intensity can reach stable state within 4 S, with a constant-current source design, the light intensity variation is within 1% after reaching the stable state. Stable light source intensity will ensure the accuracy of skin cholesterol measurement. Furthermore, after the completion of the system, we measured the repeatability of the system; the experimental results show that the coefficient of variation of high, medium and low concentration cholesterol measured by this system is not more than 5%, which will ensure the stability of skin cholesterol measurement.

We appreciate that our study uses a highly select population of subjects. Individuals with a Framingham score greater than 10% are further identified by angiography as risk groups and disease groups, which is crucial to the result because in this way we can get the accurate skin cholesterol content of the two target populations. The result of this study suggested that our device can potentially distinguish risk atherosclerosis, atherosclerosis patients and healthy individuals, as shown in Fig. 4; patients group and risk group exhibited higher skin cholesterol content than normal group. It is consistent with the previous study reported in Korea population [26], and the similar result that increased skin cholesterol can identify individuals at increased cardiovascular risk was also reported in 565 asymptomatic subjects from 6 sites in North America [27]. Dennis et al. reported that individuals with elevated skin cholesterol and elevated Framingham global risk score were fourfold more likely to have multi-vessel coronary artery disease than individuals with neither elevated [28]; our data verified the skin cholesterol of the stenosis individuals was 1.5 times higher than that of the normal subjects. However, there was no difference in skin cholesterol between the risk and disease groups; that means the level of skin cholesterol cannot distinguish two groups of people, but it still has potential applications in atherosclerosis screening. Our results first offered the relationship between skin cholesterol and atherosclerotic disease in Chinese population, which may be of great significance in the following research worldwide. With respect to patient comorbidities, we analyzed the associations between BMI, hypertension, blood glucose, total cholesterol (TC), LDL cholesterol (LDL-C), HDL cholesterol (HDL-C) and triglycerides (TG) with skin cholesterol, the result show that blood glucose, BMI, systolic blood pressure, TG and HDL-C are not related to skin cholesterol levels, only TC and LDL-C were weakly correlated with skin cholesterol content; detailed data are shown in Additional file 1: Table S2. Yashar et al. also reported there is no robust association between skin cholesterol and traditional risk factors and inflammatory markers [29]. Our clinical trials are still going on, and we will further report on the effects of medication on skin cholesterol and prospective studies of skin cholesterol in high-risk populations.

To verify the accuracy of the device, porcine skin containing gradient concentration of cholesterol was obtained using the method described in previous research [30]. As shown in Fig. 2, we detected a gradual decreased levels of cholesterol in porcine skin with the extension of extraction time. Furthermore, we compared the measurement value performed with non-invasive detection system and the gas chromatography measurement results, Fig. 3 indicated that there is a strong significant correlation between the values detected by non-invasive method and gas chromatography measured results, and the Bland–Altman analysis revealed that the Bland–Altman bias was − 72.78 ± 20.03 with 95% limits of agreement − 112.05 to − 33.51, falling within the prespecified clinically non-significant range. Both in vivo and in vitro results imply that our device is reliable and capable of identifying different amounts of cholesterol in the skin.

Like all techniques, measurement of SC with proposed method is subject to operator error. A critical step in the SC test process that is subject to operator variability is blotting, which requires the operator to remove an unbound detector from the palm before adding the indicator. Excess residual indicator solution can result in falsely increased SC levels. A simplified and standardized blotting procedure with the objective of eliminating the kind of operator variability has to be developed. As with all laboratory tests, operator training remains critical for obtaining accurate and reproducible results. One key step of the present method is that the operators of the testing institution need to be trained in strict standard procedures to complete the testing quickly and stably.

This research demonstrated the feasibility of a new system that creates a link between skin cholesterol and atherosclerosis disease. While additional experimental results and clinical data are needed to establish the reliability of this technology, the potential for atherosclerosis disease assessment and non-invasive pre-clinical atherosclerosis screening is demonstrated. However, a study from Medical University of Vienna revealed that skin tissue cholesterol concentration determined by the PREVU POC skin sterol test is not related to the presence of cerebrovascular disease (CVD) and peripheral arterial disease (PAD) or to an elevated cardiovascular risk, they also point out previous reported higher concentrations of skin cholesterol which indicated a relation between the presence of coronary heart disease (CHD) differed significantly between the studies and suffer from high standard deviations and high interquartile ranges [31]. These differences may result from pressure fluctuation and color difference in palm from different individuals conducted by different operators. Therefore, judging whether skin cholesterol can be used as a reliable indicator of atherosclerosis requires a more stable and precise approach.

This study has potential limitations. The results estimates in the research are based on prospective observational studies. Each study group included only about 100 Chinese yellow people and other skin color groups were not included. Meanwhile, none of the people included in this study had taken any lipid-lowering drugs; it is not clear whether this screening method can be applied to people who have been taking lipid-lowering drugs for a long time. Furthermore, the present study only found that people with high risk of atherosclerosis and disease had higher skin cholesterol content than normal people, but the mechanism of this phenomenon was not elucidated. Therefore, this research will focus on the following three aspects in the future: (1) multi-center clinical studies and prospective controlled trial evaluating cardiovascular disease from different races of different countries is needed to clarify the role of skin cholesterol in cardiovascular risk assessment; (2) there is an urgent need to study the effects of different types of lipid-lowering drugs, especially statins, on skin cholesterol, and to clarify whether this non-invasive screening technology can be used for long-term drug users; (3) to investigate the relationship between atherosclerosis in the coronary wall and epidermal cholesterol by combining animal models with clinical data, and to understand why skin cholesterol levels are associated with atherosclerosis.

## Conclusions

In this paper, we proposed a No-Touch palm measurement device for non-invasive skin cholesterol detection that comprises a constant-current source, a light source, a sample platform, and a spectrum detection module. Promising results from the experiments have shown that the cholesterol value measured by our device has a significant strong correlation with the results of gas chromatography, which verifies the accuracy and reliability of this technology. Meanwhile, clinical data suggest that healthy individuals and atherosclerosis patients, as well as risk atherosclerosis population can also be recognized by our device. This is the first time that the non-invasive method has been used in distinguishing risk atherosclerosis population or atherosclerosis patients from healthy individuals in Chinese population. Therefore, No-Touch palm measurement device is a promising approach for atherosclerosis risk assessment.

## Methods

### The hardware architecture and data processing algorithm of non-invasive skin cholesterol detection system

#### The hardware description of the system

**Fig. 5:**
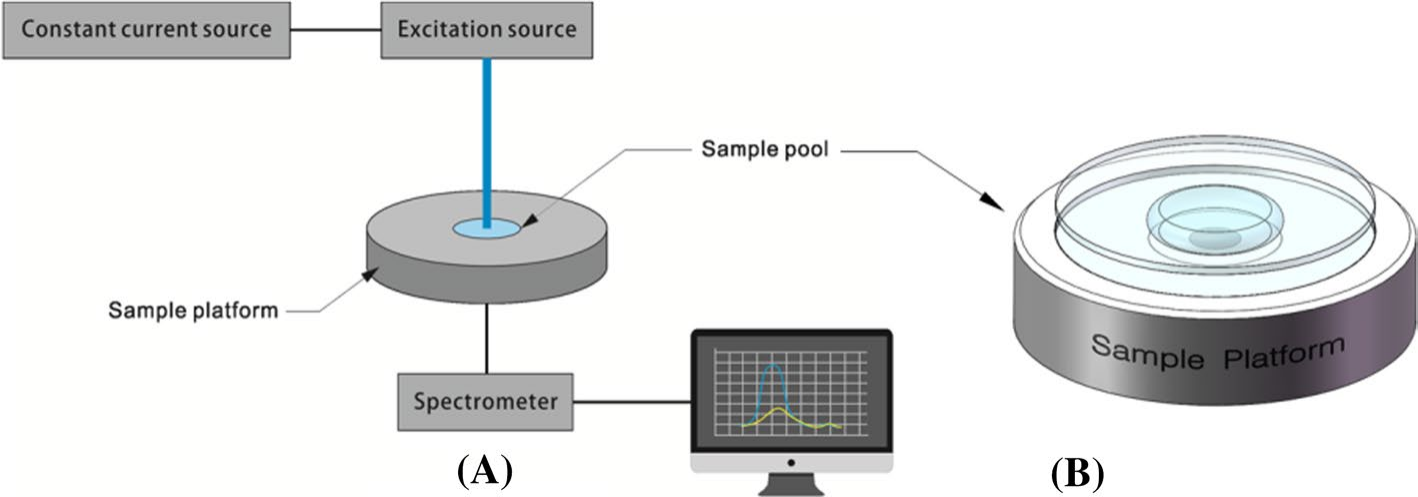
Schematic of optical system (**A**) and the structure of sample platform (**B**)

The optical system of non-invasive skin cholesterol detection system, as depicted in Fig. 5A, consists of a constant-current source module, a white LED light source (NSPL510DS, Nichia), a sample platform with a sample pool, a compact CCD device spectrometer (AM1280, OtO photonics, wavelength range is from 330 to 850 nm, resolution is 0.5 nm)and an computer. In addition, there are other parts such as power supply units, control units, optical and mechanical parts.

The white LED was chosen as the light source. The digitally adjustable constant-current source can ensure the stability of the light source. Intensity variations of the LED were typically < 1% during the measurements in the present work. Light from the LED passed through the sample, and then focused on a fiber which connected with the spectrometer. The concentration of the sample to be tested can be obtained by measuring the change in light intensity before and after through the sample.

**Fig. 6:**
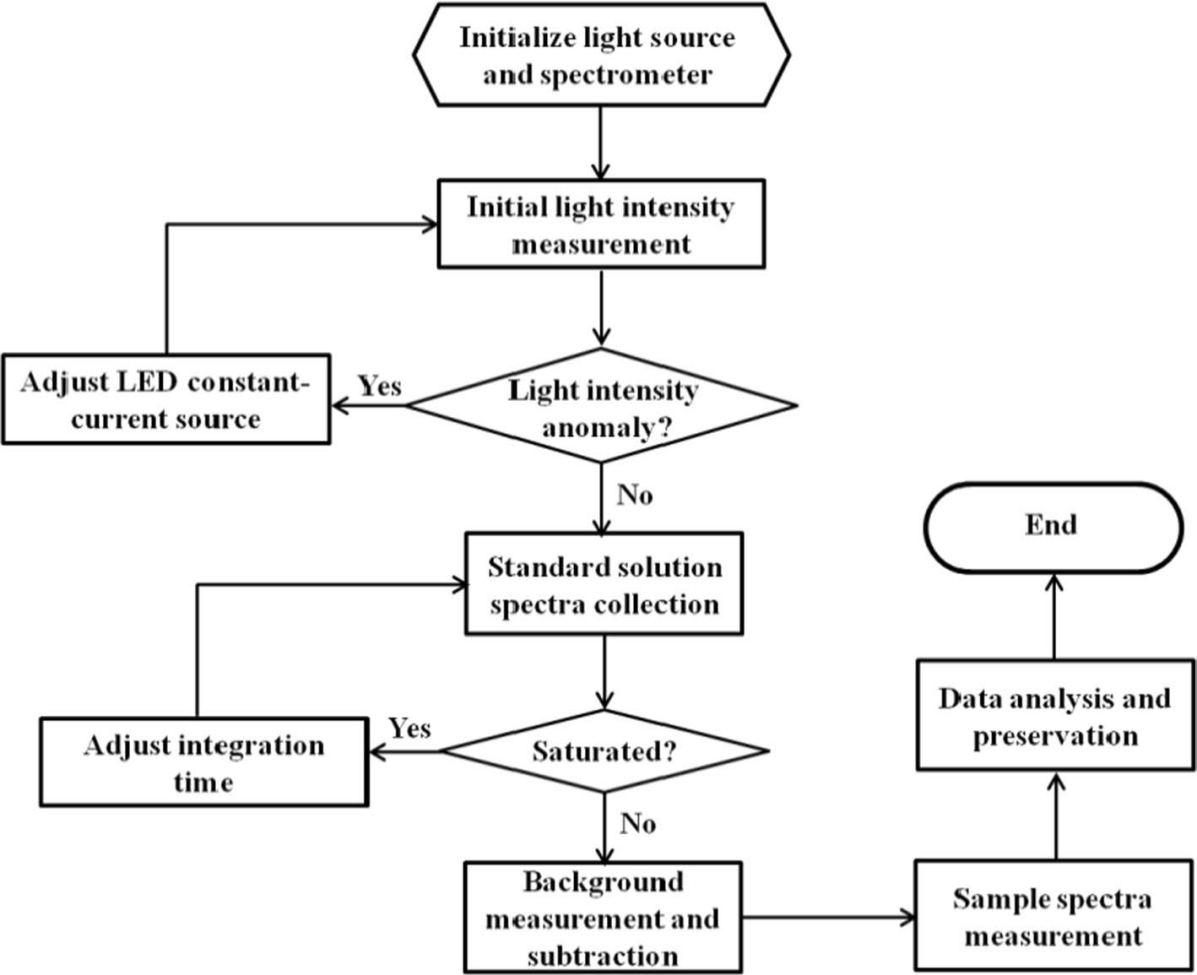
Flowchart of light source intensity control and spectra collection

**Fig. 7:**
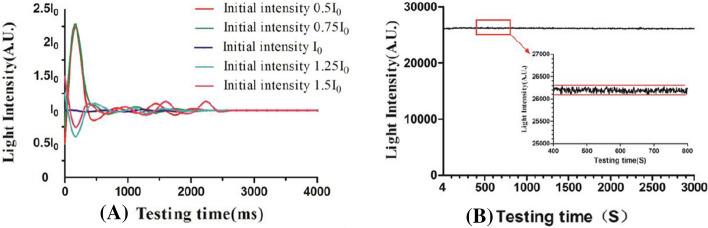
The process of LED dynamic state response (**A**) and LED steady-state response (**B**)

The stability of the light source is critical to the accuracy of the measurement. Figure 6 displays the flowchart of light source intensity control and spectra collection. Light intensity variation is within 1% by constant-current source control. In order to determine the dynamic regulation performance of the constant-current source, we tested whether the LED light source can finally reach the target light intensity under different initial light intensity, we set target light intensity as I_0_, the initial intensity varies from 0.5I_0_ to 1.5I_0_(0.5I_0_, 0.75I_0_, I_0_,1.25I_0_ 1.5I_0_), the process of LED dynamic state response curve can be obtained by constant measurement of spectrum. As shown in Fig. 7A, different initial intensity can reach target intensity, respectively, and the time reaching target intensity is no more than 4 S. Furthermore, we examined the stability of the light source after reaching target intensity, the result showed that light-intensity variation is within 1 (Fig. 7B). The above experiment results indicate that the detection system is able to reach steady light source intensity state quickly, and the intensity can also be stable for a long period of time which is essential to the accurate measurement of skin cholesterol.

When measuring the concentration of the reaction solution, we need to obtain the absorption optical path of the liquid to be tested. Therefore, it is usually necessary to put it into the cuvette, which requires a large volume of liquid. As shown in Fig. 5B, the device consists of two transparent glass sheets. The distance between the glass sheets is fixed, which reflects the depth of the detected liquid samples. The light from the light source can pass through the glass sheet and the liquid directly, and then received by the detector. Since the distance between the two glass sheets is fixed, and the absorption optical path is determined, as long as the liquid can fill the entire detection path, there is no requirement for the liquid volume. Due to the adsorption effect, only tens microliters of liquid can fill the entire detection path. Therefore, the design of the sample platform in this system requires only a small number of samples for testing.

After the completion of the system, we measured the repeatability of the system. Different concentrations of CuSO4 solution were prepared to simulate high, medium and low concentrations of cholesterol reaction solution, respectively. We choose CuSO4 solution because its color is similar to the reaction solution, and the color will not change with time at a specific concentration, which is suitable for the repeatability measurement of the instrument. The result showed that coefficient of variation (CV) was within 5%, detailed data are shown in the Additional file 1: Table S1.

### Data processing

2048 discrete data points are collected by the spectrometer with wavelengths ranging from 380 to 780 nm. After interpolation, 400 integer wavelength data are obtained. According to the absorption characteristics of the reaction solution, 475 nm to 780 nm is chosen as the characteristic band. The relative concentrations of cholesterol were retrieved based on Beer–Lambert Law, as shown in the following equation:

1 $${\mathrm{I}}_{1}\left(\uplambda \right)={\mathrm{I}}_{0}\left(\uplambda \right)\cdot \mathrm{exp}\left(-\mathrm{\alpha }\left(\uplambda \right)\mathrm{cl}\right),$$

where c is the relative concentration of the cholesterol, $\mathrm{\alpha }$ is the absorption molar extinction coefficient, and $\mathrm{l}$ is the depth of the absorber (1 mm). ${\mathrm{I}}_{0}$ is the initial light spectrum, ${\mathrm{I}}_{1}$ is the transmitted light spectrum, $\uplambda$ is the wavelength. Thus, $\mathrm{c}$ can be defined as:

2 $$\mathrm{c}=\frac{1}{\mathrm{\alpha }(\uplambda )\mathrm{l}}\mathrm{ln}\left[\frac{{\mathrm{I}}_{0}(\uplambda )}{{\mathrm{I}}_{1}(\uplambda )}\right]. $$

In the calculation process, the relative concentrations of the cholesterol sample were obtained by least squares method of the characteristic band.

### Detection steps

The detailed detection steps are as follows: first, attach the Teflon gasket with a detection well in the middle to the small thenar part of the palm. Then, add 100 μl of detection reagent to the detection well, after a 1-min incubation, the reagents are removed by blotting and the 30 μl of TMB is added to the well, after an additional 2-min incubation, 30 μl of the reaction solution is transferred to the sample pool with pipette on the sample platform. Finally, the value of cholesterol content is acquired according to the absorption spectrum of the reaction solution.

### Skin cholesterol detection reagent

The detection reagent is digitonin–copolymer–horseradish peroxidase (HRP) conjugate, which has been proposed as the specific reagent for skin cholesterol detection [15], in simple terms, digitonin have strong affinity with cholesterol, and it can combine with cholesterol to form cholesterol–digtonin complex [32–34], the copolymer ensures a tight bond between digtonin and horseradish peroxidase, and the horseradish peroxidase can catalyze the color change of 3,3’,5,5.-tetramethylbenzidine (TMB) substrate, which is dependent on the amount of cholesterol bound by the detection reagent. The reagent was synthesized following the steps described in the patent (authorization number: US05489510). TMB chromogenic reagent was purchased from Sigma-Aldrich (Shanghai, China).

### Preparation of gradient color solution

The detection reagent was diluted to 5 μg/ml, 2.5 μg/ml, 1.25 μg/ml, 0.625 μg/ml, 0.3125 μg/ml and 0.15625 μg/ml with deionized water to catalyze with TMB, and the gradient color solution is prepared, the amount of colored products produced by the reaction was measured with our system.

### Gradient cholesterol extraction from porcine skin

Abdominal skin samples were obtained from six Tibetan pigs weighing 30–35 kg, and were provided by the animal center of Anhui Medical University. Two reasons led us to choose pig skin as the experimental sample, firstly, the epidermis of both human and pig have similar structure and thickness, the most obvious similarity is that pigs and humans share the characteristic of having sparse body hair. This is particularly important, as hair follicles together with their sebaceous glands have been recognised as pathways for percutaneous penetration of topically applied drugs and some chemical substances, and the pig skin is a common choice to simulate human skin. Secondly, for an animal 5 pieces of skin tissue with the size of 1.5* 7.5 cm are needed for the experiment; the body surface area of mice, rats and guinea pigs is not large enough for one experiment. Normal saline (shanghai, sigma) was used to soak the skin after subcutaneous tissue was removed so as to exclude hemoglobin and other pollution. The skin was then dried in the shade of the nature after cutting into 1.5*7.5 cm rectangular cubes. Skin cholesterol was extracted afterward in the mixture of ethanol and ethyl ether with a proportion of 3:1 for different time course (0 min, 1 min, 2 min, 3 min and 4 min). The cholesterol in the epidermis can be dissolved by the mixed solvent. With the extension of time, the remaining cholesterol in the epidermis will be less and less. In this way, the skin samples containing different levels of cholesterol can be obtained.

### Participants

342 participants were enrolled consecutively from two sites, 232 individuals were recruited in Health Management Center of Renmin Hospital of WuHan University, 110 patients with overt vascular disease according to the angiogram were collected from the First Affiliated Hospital of University of Science and Technology of China, Exclusion criteria included (a) current lipid-lowering therapy or lipid-lowering therapy within the last year; (b) age less than 18 years; (c) pregnancy; (d) psoriasis or eczema on either hand; (e) recent use (within 24 h before testing) of topical medication, as a cream or lotion; (f) chronic liver disease or evidence of abnormal liver function; (g) conditions that might lead to an incomplete follow-up (i.e., life expectation 6 months). Everyone involved in the research had skin cholesterol measured and baseline risk data recorded. Antecubital venous blood samples were obtained for the determination of total plasma cholesterol (TC), serum low-density lipoprotein cholesterol (LDL-C), high-density lipoprotein cholesterol (HDL-C), triglycerides (TG), glucose levels. The Framingham risk score (FRS) is a simplified and common tool for the assessment of risk of coronary artery disease (CAD) over 10 years, and it is the most applicable method for predicting the person’s chance of developing cardiovascular disease (CVD) in the long term. Because this risk score gives an indication of the likely benefits of prevention, it can be useful for both the patients and clinicians deciding whether lifestyle modification and preventive medical treatment. Absolute CVD risk percentage over 10 years was classified as low risk (< 10%), intermediate risk (10–20%), and high risk (> 20%). Of the 232 subjects recruited in Health Management Center of Renmin Hospital of WuHan University, 115 individuals had a score < 10% and 117 subjects with no obvious stenosis of the vessel had a score ≥ 10%. All 110 patients with overt vascular disease had a score > 10%, overt vascular disease was defined as (a) stenosis of at least 50% in at least 1 vessel(any disease) and (b) stenosis of at least 50% in ≥ 2 vessels (multi-vessel disease). The main purpose of this clinical study is to explore the difference of skin cholesterol content among low-risk group, risk group (intermediate risk and high risk) and patients with cardiovascular disease. Therefore, the participants were divided into normal group, risk group and disease group, the normal group was with a FRS < 10%, the risk group was with a FRS ≥ 10% and no vascular stenosis, and the disease group was with a FRS ≥ 10% and overt vascular disease. Meanwhile, another 72 volunteers were recruited to participate in the accuracy verification, briefly, everyone involved in this experiment would measure skin cholesterol with non-invasive detection system, then the detection site will extracted with 400 ul of absolute ethanol for 2 min, cholesterol in extractive liquid were determined with gas chromatography immediately. Technicians at each site were trained in an identical manner to measure skin cholesterol. The study protocol was approved by the local ethics committee, and written informed consent was obtained from all patients.

### Cholesterol measurement with gas chromatography

Cholesterol standard (Sigma-Aldrich) is dissolved in absolute ethanol to the concentration of 1 ug/ml, 2 ug/ml, 5 ug/ml, 10 ug/ml, 25 ug/ml and 50 ug/ml. Gas chromatography is used for detection of cholesterol content followed the previously reported method [35], briefly, detection condition is as follows: column, DB-5 elastic quartz capillary column. Carrier gas, high-purity nitrogen, purity ≥ 99.999%; constant flow rate, 2.4 mL/min; column temperature (programming temperature): initial temperature is 200° C, hold for 1 min, increase to 280° C at 30° C / min, for 10 min; inlet temperature, 280 °C; detector temperature, 290 °C; injection volume: 1ul; injection method: no split injection, open the valve after 1 min of injection; Air flow: 350 mL/min; hydrogen flow rate: 30 mL/min. The cholesterol standard solution was separately injected into the gas chromatograph, and the peak area of the standard solution was measured under the above chromatographic conditions, and the standard curve were prepared by taking the concentration as the abscissa and the peak area as the ordinate. Then the extract was injected into the gas chromatograph to measure the peak area, and the concentration of cholesterol in the sample solution was obtained from a standard curve.

### Statistical analysis

One-way ANOVAs (Graphpad, Prism 5) were utilized for multiple-group comparisons. Correlations of skin cholesterol content measured by gas chromatography with non-invasive detection method were investigated using linear correlation analysis. Bland–Altman analysis was used to investigate the difference assessment between the non-invasive detection method and the gas chromatograph method. The area under the receiver-operating characteristic curve was applied to evaluate the diagnostic efficacy of non-invasive detection method. All analysis was presented as means ± SD, A P value < 0.05 was considered statistically significant.

## Supplementary Information

**Additional file 1: Table S1.** Coefficient of variation of the system measured with different concentration of CuSO4. **Table S2** Physiologic correlation of skin cholesterol measurement as assessed by univariate analysis.

## Data Availability

All data used and analyzed during the study are available from the leading corresponding authors Meili Dong and Yikun Wang (wyk@aiofm.ac.cn; dongmeili@aiofm.ac.cn) on reasonable request.

## Abbreviations

SC: Skin cholesterol
CIMT: Carotid intima–media thickness
HRP: Digitonin–copolymer–horseradish peroxidase
TMB: 3,3’, 5,5'-Tetramethylbenzidine
LED: Light emitting diode
CCD: Charge-coupled device
TC: Total cholesterol
LDL-C: Low-density lipoprotein cholesterol
HDL-C: High-density lipoprotein cholesterol
TG: Triglycerides
