# Supplementary material for: Development of a non-invasive method for skin cholesterol detection: pre-clinical assessment in atherosclerosis screening
Source: Biomed Eng Online. 2021 Jun 1;20:52. doi: 10.1186/s12938-021-00889-1 (PMC8170999; doi:10.1186/s12938-021-00889-1)
Supplement: Supplementary file 1 — Additional file 1: Table S1. Coefficient of variation of the system measured with different concentration of CuSO4. Table S2 Physiologic correlation of skin cholesterol measurement as assessed by univariate analysis. [file 12938_2021_889_MOESM1_ESM.docx]

**Supplementary material**

**Table1. Coefficient of variation of the system measured with different concentration of CuSO4**

| **Concentration of CuSO4(mg/ml)** | **Coefficient of variation(CV)(%)** |
| --- | --- |
| 20 | 4.3% |
| 100 | 3.5% |
| 500 | 3.7% |

**Table 2. Physiologic correlation of skin cholesterol measurement as assessed by univariate analysis**

|  | **Correlation coefficient** | **P value** |
| --- | --- | --- |
| BMI | 0.39 | 0.18 |
| Blood glucose | 0.38 | 0.23 |
| Systolic blood pressure | 0.41 | 0.25 |
| TC (mmol/L) | 0.55 | 0.04 |
| LDL-C (mmol/L) | 0.48 | 0.02 |
| HDL-C (mmol/L)  TG | 0.51  0.42 | 0.07  0.16 |
